# Kinematic Effects of Derotational Osteotomy of the Humerus in Patients with Internal Shoulder Contracture Secondary to Erb’s Palsy—A Retrospective Cohort Study

**DOI:** 10.3390/jcm13102759

**Published:** 2024-05-08

**Authors:** Anna-Lisa Pignet, Andreas Kranzl, Andrzej Hecker, Gerlinde Weigel, Lars-Peter Kamolz, Werner Girsch

**Affiliations:** 1 Division of Plastic, Aesthetic and Reconstructive Surgery, Department of Surgery, Medical University of Graz, 8036 Graz, Austriawerner.girsch@kabsi.at (W.G.); 2 Laboratory for Gait and Movement Analysis, Orthopaedic Hospital Vienna-Speising, 1130 Vienna, Austria; 3 Austrian Armed Forces, Medical Center East, Medical Facility Vienna, 1210 Vienna, Austria

**Keywords:** obstetric brachial plexus palsy, humerus derotation osteotomy, three-dimensional motion-capture

## Abstract

**Background:** Internal rotation contractures of the shoulder are common sequelae of conservatively treated obstetric brachial plexus palsy (OBPP) with incomplete spontaneous neurological recovery. Humerus derotation osteotomy has been suggested as a possible treatment option to improve arm positioning. However, consensus as to whether humerus derotation osteotomy can successfully restore limb function is missing. **Methods:** In the present controlled cohort study, we aimed at analyzing global upper extremity kinematics with a 3D-video analysis system in children with shoulder internal rotation contractures secondary to OBPP before, and one year after, humerus derotation osteotomy. Patients under 18 years of age that presented to our center with conservatively treated internal rotation contractures of the shoulder and subsequently underwent humerus derotation osteotomy were included. The unimpaired arm served as a respective control. **Results:** Pre-operatively, all patients showed severe internal rotation contractures of the shoulder of almost 60° at rest. At the follow-up, the position of the shoulder at rest was greatly shifted to 9° of internal rotation. The patients showed statistically significant improvement in maximum external rotation and abduction of the shoulder, as well as in maximum flexion of the elbow, and the range of motion of pro/supination. The maximum internal rotation of the shoulder, however, was diminished after the osteotomy. **Conclusions:** Our data indicated that derotational osteotomy is a promising procedure which can be used to correct for internal rotation contractures secondary to OBPP. Moreover, 3D-video analysis proved to be a useful tool that supplies the surgeon with both precise information about the degree of distortion pre-operatively, thus helping to decide on the amount of correction, and secondly, a measurement of the post-operative gain in upper extremity function.

## 1. Introduction

Erb’s palsy, or upper obstetric brachial plexus palsy (OBPP), is defined as an injury to the upper part of the brachial plexus that occurs during delivery [1]. Despite a relatively high spontaneous recovery rate, the incidence of persisting OBPP is still at 0.46 out of 2.9 affected live births per 1000 [2]. If left untreated, affected patients face permanent limitations in upper extremity (UE) function with deficits in external rotation, abduction of the shoulder and sometimes a weakness of forearm rotation [3,4]. Later on, affected patients commonly develop internal rotation contractures of the shoulder, and sometimes even elbow flexion contractures, leading to serious difficulties in performing various tasks of daily living. Hoeksma et al. found that more than half of OBPP patients with shoulder contractures show an extension deficit of more than ten degrees [5]. The prevalence of elbow flexion contractures varies from 48% to 85%, depending on the population studied [3,6]. At that stage, only secondary reconstructive procedures, such as muscle releases, tendon transfers, and humerus derotation osteotomy can be performed [7,8]. Al-Qattan et al. stated that derotation osteotomy improves cosmetic appearance, rather than limb function [9]. Waters et al., on the other hand, showed a significant improvement of global shoulder function after humerus derotation osteotomy [10]. Derotation osteotomy surely allows for a better positioning of the arm; however, consensus on whether it can successfully restore limb function is missing [9,10]. So is objective, unbiased, and recent data. While visual estimation of the range of motion (ROM) of elevation of the shoulder is somewhat reproducible (if performed by trained primary care physicians), this is not the case with external rotation of the shoulder [11]. The struggle to obtain meaningful data is further increased when it comes to the examination of fidgety infants. A tool to overcome these issues has for many decades successfully been used for lower limb analysis, namely optoelectronic three-dimensional (3D)-video analysis [12]. This is a promising method for objective, quantitative assessment of movements in all degrees of freedom [13,14,15,16]. However, compared to gait analysis, scientific attention on motion analysis of the UE is sparse [15,17]. Because of the more complex nature of the upper limb, especially at the shoulder girdle, and the great variability of possible movements, the transfer from the lower extremities poses a challenge. Simplifications concerning the biomechanical model have to be taken into account to find a compromise between accuracy and practicability for clinical applications [17]. Prior studies in the field have been performed to investigate the physiological range of motion in healthy adult and pediatric populations and also to capture limitations in different entities of upper extremity impairment, such as OBPP, hypertonia due to secondary dystonia, or hemiplegic cerebral palsy [18,19,20,21,22,23].

To our knowledge, optoelectronic 3D-video analysis has not yet been introduced for the evaluation of UE surgical outcomes. In this cohort study, we aimed at the objectification of global UE kinematics in children with internal rotation contractures as sequelae to Erb’s palsy. The unaffected arms served as respective controls. Then, we also aimed to evaluate the kinematic improvements in a one-year follow-up after humerus derotation osteotomy, based on a biomechanical model which is presented here.

## 2. Materials and Methods

The retrospective monocentric cohort study was conducted in the Gait and Motion Analysis Laboratory of the Orthopaedic Hospital Speising-Vienna, Austria, and approved by the ethics committee of the city of Vienna (EK 14-272-VK, 22 January 2015).

### 2.1. Patients

We included all patients below 18 years of age that presented to our center with internal contractures of the shoulder due to persisting Erb’s palsy, and who were operated on for humerus derotation osteotomy by the senior author. The exclusion criterion was an absence of kinematic data. 

### 2.2. Surgical Protocol for Internal Contractures Due to Erb’s Palsy

At our center, all patients above three years of age that presented with internal contractures of the humerus due to Erb’s palsy and the wish for functional improvement were treated with secondary reconstructions. The chosen surgical sequence relied on the severity of distortion. In milder cases that predominantly lacked external rotation, solely humerus derotation osteotomy was performed. In the presence of an additional abduction deficit (60–90° maximum abduction), an additional release of pectoralis major and subscapularis muscles was performed. In case of a severe abduction deficit (30–60° maximum abduction), a three-step surgical procedure including humerus derotation osteotomy, muscle release, and a triple muscle transfer were performed to adequately restore limb function. For the muscle transfer, the latissimus and teres major muscles were sutured into the infraspinatus tendon, while the levator scapulae muscle was transferred to the supraspinatus tendon. To ensure adequate rotation of the humerus during derotation osteotomy the shoulder was intraoperatively passively adducted until the hand would passively reach the abdomen. Then the shoulder was passively abducted until the hand would reach the patient’s face and the back of the head. The derotated position was secured with clamps until the correct degree of external rotation was found, which amounted to 30° in most cases. Once an adequate rotation was ensured, an osteosynthesis plate was fixed with screws to the proximal segment in classic compression osteotomy style. A cast was applied for six weeks, after which children were allowed to use the limb freely.

### 2.3. Biomechanical Model for Motion-Capture Analysis

This study used a modified 3D motion-capture system, similar to the system described by Rab et al. [16]. The system we used (available from Motion Analysis Corporation of Santa Rosa, CA, USA) was equipped with six infrared cameras. To increase accuracy, 27 instead of 18 retro-reflective markers were attached to bony landmarks. The light source located in the cameras tracked the displacement of the passive reflective markers located at specific anatomic landmarks. Data were sampled at a rate of 60 Hz. The software required for the identification of the tracked markers was called Motion Analysis Expert Vision A (Motion Analysis Corporation, Santa Rosa, CA, USA). To quantify joint deflections in degree (°) and movement velocities in degrees/ms by regarding anatomic axes and joint centers, the software components Eva 6 and Ortho Trak 6.33 (Motion Analysis Corporation, Santa Rosa, CA, USA) were used. The biomechanical model was a rigid model, similar to the one described by Rab et al. It consisted of four segments: the shoulder girdle, the upper arm, the lower arm, and the wrist [16]. For ease of calculation, all joints were assumed to have fixed centers of rotation. The shoulder joint was modeled as a ball and socket joint with three degrees of freedom. We recorded a combined movement of the glenohumeral and the scapulothoracic articulations [16,24]. As the motion-capture system calculates angles with respect to the segment coordinate system, lower values for joint deflection are calculated than visually estimated, especially at the shoulder girdle. Physical examinations of the shoulder girdle using the goniometer include to a certain degree the trunk’s contribution to the gesture, while values obtained from motion analysis exclude the support of these anatomic units; e.g., shoulder abduction, which actually occurs in the glenohumeral joint, is supported by rotation of the scapula (scapulothoracic movement), the elevation of the ipsilateral shoulder girdle in the sternoclavicular joint, and by lateral tilting of the spine to the contralateral side. Apart from scapula rotation, the system calculates angles for each segment separately. Another example is shoulder internal rotation. This movement is not only performed in the glenohumeral joint, but also supported by anteversion of the shoulder girdle, which cannot be included in the analysis either. 

The elbow joint, however, was modeled as a rotating hinge joint with two degrees of freedom, with a single joint center in the distal humerus. This is another simplification, as the pronation and supination of the forearm are not only performed in the elbow, but in the proximal and distal radio-ulnar joints [25]. Wrist movement is characterized by movement between the hand and forearm segments, determined by a vector connecting the geometric wrist center and the calculated elbow center [16,24,26].

### 2.4. Marker-Setup across Distinct Anatomic Landmarks

**Figure 1:**
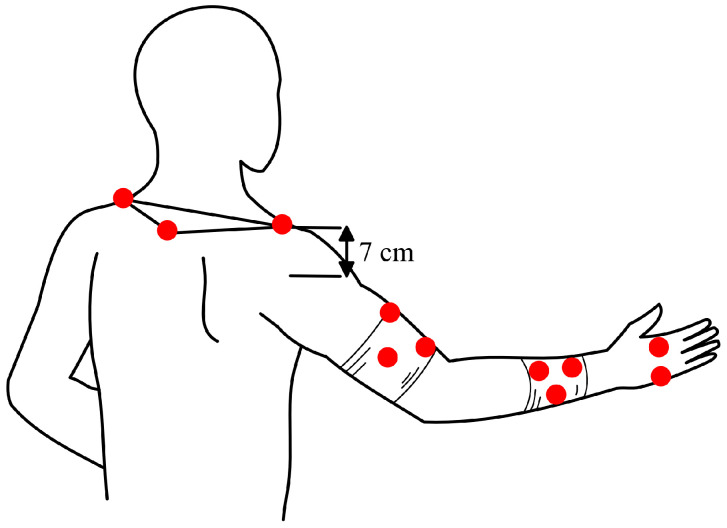
Illustration of the placement of the retroflective markers (red dots) on the upper extremity.

On each body segment a minimum of three non-collinear markers were placed to fully define its motion in all three planes. We used rigid triads of markers in order to suppress inter-marker motions due to skin movements [15]. As it is unfeasible to place markers inside joints, their location was mathematically translated from their actual positions to the joint. Markers were attached to upper arm and forearm segments and additional markers to the lateral and medial epicondyle and to the distal ends of the radius and ulna, lateral, and medial to the wrist flexion axis (Figure 1). The joint center of the wrist was assumed to be the midpoint between the ulnar and radial wrist markers. The elbow joint center was calculated as the midpoint between the medial and lateral elbow markers. During the static reference measurement, the subject remained in a relaxed, upright position, with the elbow slightly flexed, the upper-arm vertical, and the wrist straight [27]. After the static reference measurement, markers for elbow and wrist joint calculations had to be replaced, as the actual joint position of the elbow and wrist were calculated from the triangles of the upper and lower arm during motion analysis. Further markers were added to define the shoulder girdle, the shoulder joint, and the wrist. The plane of the shoulder girdle was determined by two markers, one placed on the processus spinosus of C7 and another fixed to the left and right acromion. The shoulder center was assumed to be seven centimeters inferior to the acromion marker, which is the average of visually determined distances using a ruler. Finally, two markers were attached to the dorsum of the hand to define the most distal segment of our biomechanical model. Thumb and finger movements were not included in the analysis.

### 2.5. Measurement Procedure for Selected Single Movements and Movement Velocity

We propose a sequence of active single movements, for a straightforward pre- and post-surgical comparison, and to allow precise pre-operative planning. At the start of each trial, the joint deflection of the shoulder, elbow, and wrist in the position at rest were captured. The position at rest was defined as an upright position where patients felt most comfortable with their arms at their sides and with forearms naturally pronated. Starting from this position, the patients were instructed to carry out different types of active single movements. In the shoulder joint, these included the following: abduction, adduction, internal and external rotation, retroversion, and anteversion. In the wrist and forearm, we measured pronation and supination, as well as elbow flexion and extension. When the subjects were asked to flex the elbow, we simultaneously captured the trunk movements. During abduction of the shoulder and flexion of the elbow, the velocities of movement execution were simultaneously recorded in degrees/ms. Every time the maximum joint deflection was reached, the limb was returned into the position at rest. 

In the group of patients, the whole sequence was performed twice, each time with both the impaired and the unimpaired limbs. The unimpaired limb served as control, as proposed by Wang et. al, who found no significant differences between UE kinematic of a healthy population and the unimpaired arm of children with OBPP [28]. All patients performed the selected sequence before and 12 ± 2 months after humerus derotation osteotomy.

### 2.6. Statistical Analyses

Statistical analyses were performed using Prism 9.3.1 (GraphPad Software, LLC, San Diego, CA, USA). The analysis involved calculating the median and range. Apart from descriptive statistics, a Wilcoxon matched-pairs signed rank test was conducted to compare the affected and the unaffected limb pre-operatively, as well as the affected limb pre- and post-operatively, within the group of patients. In addition, the unaffected limb pre-operatively was compared to the unaffected limb post-operatively. All *p*-Values < 0.05 were considered statistically significant.

## 3. Results

### 3.1. Patient Characteristics

We were able to include twelve patients who underwent derotation osteotomy of the humerus due to internal shoulder contractures, with a mean age of 9 years (range: 5–12 years). Seven were males and five, females (Table 1). None of the patients received any prior surgical interventions regarding their OBPP. Three patients (P2; P8; and P11) underwent humerus derotation osteotomy only, four patients (P1; P3; P4; and P9) underwent an additional muscle release, and five patients (P5; P6; P7; P10; and P12) were operated on for humerus derotation osteotomy, muscle release, and a triple muscle transfer (Table 1). Eleven out of twelve patients were available for pre- and post-surgical 3D-video analysis. In one patient, we obtained post-surgical data only. The same hand surgeon (W.G) operated on all of them. At the time of the follow-up (12 ± 2 months after surgery), no cases of non-union nor any adverse or unanticipated events were documented.

### 3.2. Pre- and Post-Operative Global Upper Extremity Kinematics

#### 3.2.1. Persisting OBPP Leads to Kinematic Restrictions of the Entire Upper Limb

**Figure 2:**
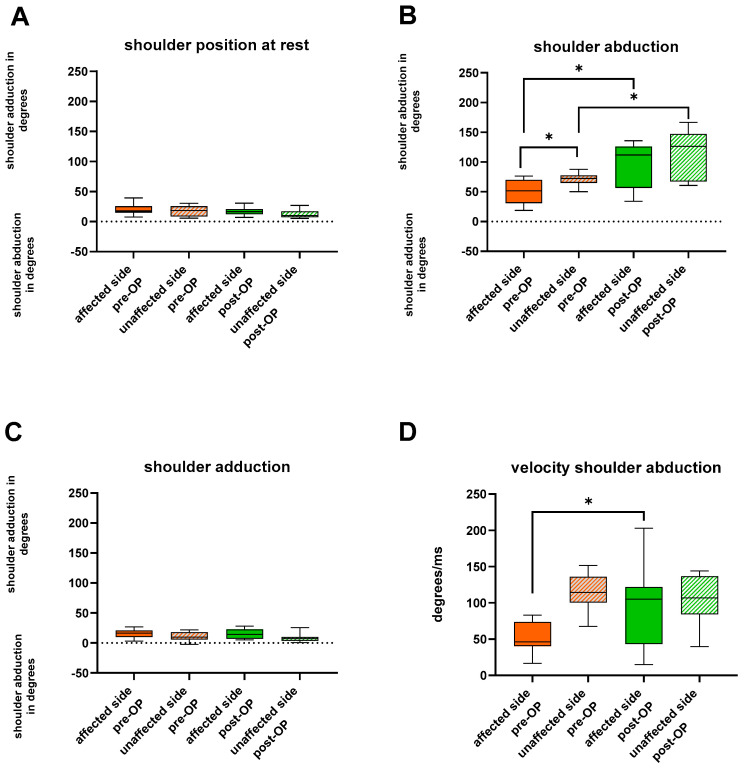
(**A**) shows box plots depicting angular degrees of abduction and adduction of the shoulder in the position at rest of the affected and unaffected limb both pre− and post−operatively. (**B**,**C**) show the angular degrees of abduction and adduction when patients were asked to perform (**B**) maximum abduction and (**C**) maximum adduction of the shoulder. (**D**) depicts the velocity of abducting the shoulder in degrees/ms achieved with the affected and unaffected limb both pre− and post−operatively. The whiskers depict the extent of the minimum and maximum values. * *p* < 0.05.

**Figure 3:**
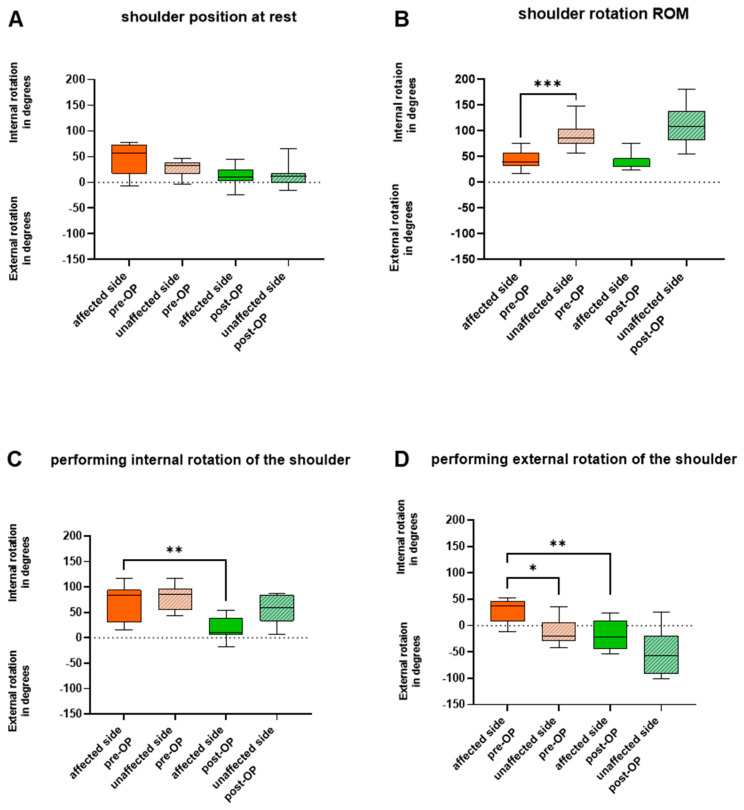
(**A**) depicts the angular degrees of shoulder rotation in the position at rest. (**B**) illustrates the ROM of shoulder rotation. (**C**,**D**) both show the maximum joint deflection achieved with the affected and unaffected limb pre− and post−operatively when patients were asked to perform (**C**) internal rotation and (**D**) external rotation of the shoulder. The whiskers depict the extent of the minimum and maximum values. * *p* < 0.05; ** *p* < 0.01; *** *p* = 0.001.

Pre-operatively, the affected shoulder was positioned in an internal rotation angle of 58° at rest, compared to 33° on the unaffected counterpart (Figure 2). External rotation was unfeasible on the affected side. In an attempt to perform external rotation of the shoulder, the internally rotated shoulder merely shifted to a lower internal rotation angle (from 57° to 37°). With the unaffected limb an external rotation angle of 19° (*p* = 0.0322) was achieved. Maximum internal rotation, on the other hand, did not reveal significant pre-operative changes between the two sides (Figure 3). The angle of shoulder abduction was again significantly decreased on the affected side (52° vs. 73°) (*p* = 0.0244), while shoulder adduction did not differ (Figure 2). We also found restrictions in anteversion (*p* = 0.0137), but not in retroversion of the shoulder. 

In the elbow, the position at rest was pre-operatively severely flexed (flexion angle of 31). Elbow mobility was also significantly limited; extension was lacking (flexion angle of 18°), and maximum flexion (122°) was restricted, compared to the healthy limb (138°) (*p* = 0.0322; *p* = 0.0244) (Figure S1). The median pre-operative ROM for pro/supination was significantly reduced on the affected side (37° vs. 75°) (*p* = 0.0024) (Figure S2). Results as to thoracic mobility are illustrated in Figure S2. All pre- and post-operative values are illustrated in Table 2.

#### 3.2.2. Humerus Derotation Osteotomy Addresses Global Upper Extremity Kinematics

At the follow-up, patients attained a resting posture of 9° of internal rotation of the shoulder. Compared to the pre-operative values the amount of internal rotation of the shoulder at rest decreased significantly (*p* = 0.0186). Simultaneously, the maximum internal rotation of the shoulder was also significantly decreased from a median of 84° to 10° (*p* = 0.0098). Maximum external rotation of the shoulder, on the other hand, greatly increased to 21° (*p* = 0.0068). Shoulder abduction and motion velocity were post-operatively doubled (111.8°; 104.9°/ms) (*p* = 0.0322). We found no significant changes in anteversion and retroversion of the shoulder. 

Elbow flexion was successfully restored to 137° (*p* = 0.002), whereas velocity of elbow flexion, elbow extension, and elbow positioning at rest showed no significant changes. Pro/supination were also significantly increased to a ROM of 69° (*p* = 0.0391) at the time of the follow-up. Interestingly, pre- and post-operative values for some motions of the unaffected side differed significantly; namely, shoulder abduction (*p* = 0.0195), elbow flexion (*p* = 0.0137) and extension (*p* = 0.0098), and pro/supination (*p* = 0.0098). All *p*-values, as well as pre- and post-operative values, are illustrated as the median and range in Table 2.

## 4. Discussion

Motion analysis of the upper extremity provides extensive kinematic information. We showed that several different parameters besides maximum joint deflection can be expressed per joint. What is more, motion analysis facilitates the analysis of complex and composite movements. It allows for the separate analysis of motions, such as thoracic movements during elbow flexion, and captures ROMs that are otherwise difficult to objectively measure, such as shoulder rotation. If performed prospectively, it allows the surgeon to plan the upcoming treatment more carefully [15]. The recording of single movements created joint angles in degrees, which are crucial for accurate pre-operative planning. However, the incorporation of activities of daily living might be beneficial to evaluate the functionality of the respective limb in daily tasks [19]. 

In the group of Erb’s palsy patients, we showed that due to the internal rotation contracture and the consequent unfavorable positioning of the arm, not only external rotation, but global UE kinematics were restricted. Restricted movements (compared to the unimpaired counterpart) besides external rotation included shoulder abduction and elbow flexion, as well as pronation and supination. In addition, we found that when performing elbow flexion with the affected limb, patients simultaneously tended to lean their torso backwards (thoracic flexion). The follow-up data after secondary reconstruction including humerus derotation osteotomy revealed that most of the kinematic restrictions were successfully improved. Movements that improved most were external rotation and abduction of the shoulder, elbow flexion, and pro/supination. The angular degrees of these motions reached values similar to the unimpaired limb. A significant increase was also found in the velocity of shoulder abduction. Even though other authors found no difference in forearm movements in children after shoulder procedures, our patients showed significant improvements [3]. Hence, maximum elbow flexion was increased, despite not being surgically addressed. This improvement might be due to the altered positioning of the arm, which seemed to equally affect forearm and elbow kinematics. Only one single movement was found to be post-operatively reduced in maximum joint deflection, namely, the internal rotation of the shoulder. Hence, angular degrees for internal rotation were lower than before surgery and lower than in the control arm, which indicated an overcorrection of external rotation of the shoulder. The restricted ability to perform internal rotation could cause difficulties in reaching the center of the body and performing daily actions such as buttoning-up trousers. Due to these results, we were reconsidering the amount of correction (about 30°) in the common practice of humeral derotation osteotomy. Surprisingly, the unaffected side showed significant post-operative increases in shoulder abduction, elbow flexion, extension, and pro/supination. As the follow-up was conducted one year later, this might have come about by a training effect or in the maturation of UE movement. UE movement maturation is thought to reach a plateau around the age of 11 years [29]. An error in marker placement or identification cannot be ruled out, but this seems rather unlikely due to the high reproducibility. 

## 5. Limitations

We acknowledge the small sample size, which is certainly prone to outliers, as well as the heterogeneity in our study population. In the future, a more sophisticated model could be implemented to take the complexity of the shoulder girdle into account, as consisting of two separate articulations. We refrained from doing that, as accurate determination of scapular position remains difficult without time-consuming palpation or imaging techniques.

## 6. Conclusions

In conclusion, humerus derotation osteotomy seems indeed to successfully restore solid upper limb function, predominately targeting shoulder abduction, external rotation and pro/supination. However, care should be taken to avoid overcorrection of external rotation in the shoulder. 3D-video analysis allows for an objective documentation of pre- and post-surgical changes of complex UE and trunk movements. The acquired kinematic data help the surgeon to plan upcoming procedures more carefully, to scrutinize the chosen treatment option, and to improve current concepts for future cases. 

## Figures and Tables

**Table 1:** Patient characteristics and type of surgery.

Patients	Sex	Age (Years)	Affected Arm	Surgery	Complications
1	M	9	Left	2	None
2	F	10	Left	1	None
3	F	8	Right	2	None
4	F	9	Right	2	None
5	F	8	Left	3	None
6	F	8	Right	3	None
7	M	13	Right	3	None
8	M	7	Left	1	None
9	F	10	Right	2	None
10	M	5	Left	3	None
11	M	10	Right	1	None
12	F	8	Left	3	None

Among the included twelve patients for kinematic data analysis, seven were females (F) and five were males (M); all were aged 5–13 years at the time of surgery. Half of them underwent surgery at the left limb and half at the right. The respective type of surgery is encoded with 1–3; 1: humerus derotation osteotomy only, 2: additional muscle release, 3: humerus derotation osteotomy, muscle release and triple tendon transfer. F: females; M: males.

**Table 2:** Pre- and post-operative data of upper extremity kinematics of the affected and unaffected limb.

Median (Range)	1. Affected Pre-OP	2. Unaffected Pre-OP	3. Affected Post-OP	4. Unaffected Post-OP	*p*-Value (1 vs. 2)	*p*-Value (1 vs. 3)	*p*-Value (3 vs. 4)	*p*-Value (2 vs. 4)
Shoulder internal rotation at PAR	57.6 (−7.1–77.1)	32.9 (−4.1–45.8)	9.3 (−24.5–44.9)	12.31 (−16.2–66.2)	0.1934	0.0186	0.1953	0.20
Shoulder internal rotation	84.4 (15.5–116.3)	84.7 (43.2–117.6)	9.9 (−17.1–54.1)	58.8 (7.2–80.4)	0.5771	0.0098	0.010	0.1289
Shoulder external rotation	37.2 (−12.0–52.3)	−19.4 (−42.7–35.0)	−20.9 (−53.9–23.6)	−57.5 (−100.6–25.7)	0.0322	0.0068	0.01	0.0547
Shoulder rotation ROM	39.0 (17–76)	86.0 (56–147)	31.0 (24–75)	108.0 (54–181)	0.0010	0.6172	0.002	0.30
Shoulder abduction at PAR	17.8 (7.5–39.5)	18.4 (5.8–30.4)	16.6 (6.9–39.6)	10.1 (5.4–27.9)	0.5566	0.4316	0.99	0.20
Shoulder adduction	16.7 (3.2–26.7)	10.0 (−2.1–22.1)	14.4 (5.2–28.3)	7.4 (0.9–25.6)	0.1230	0.8311	0.006	0.43
Shoulder abduction	51.7 (18.6–76.3)	72.5 (50.0–87.7)	111.8 (34.2–135.9)	126.5 (60.8–166.5)	0.0244	0.0322	0.002	0.0195
Shoulder abduction velocity (*)	46.2 (16.7–82.9)	114.4 (67.4–151.4)	104.9 (14.8–188.0)	106.9 (39.4–144.1)	0.0020	0.0273	0.16	0.8203
Shoulder anteversion	91.3 (31.9–136.4)	114.5 (92.4–150.2)	117.0 (46.3–143.3)	148.7 (106.6–171.6)	0.0137	0.0645	0.004	0.02
Shoulder retroversion	14.5 (−19.7–44.0)	1.3 (−25.3–51.0)	−3.9 (−44.1–14.9)	−11.0 (−157.1–18.5)	0.2334	0.1230	0.76	0.15
Elbow at PAR	31.4 (15.7–43.5)	10.8 (7.0–43.8)	39.2 (22.8–77.3)	34.3 (9.2–53.2)	0.0420	0.0645	0.010	0.003
Elbow flexion	122.2 (56.1–132.3)	138.1 (105.5–147.1)	137.4 (121.5–158.3)	150.8 (132.0–161.4)	0.0244	0.0020	0.02	0.0137
Elbow extension	17.6 (−53.7–91.6)	−2.1 (−61.0–29.7)	38.8 (13.9–75.9(	28.7 (−7.5–47.4)	0.0322	0.1602	<0.001	0.0098
Elbow flexion velocity (*)	114.9 (55.4–185.4)	117.2 (66.4–202.6)	106.9 (66.8–252.3)	172.5 (108.4–196.6)	0.91	0.8203	0.05	0.0547
Wrist at PAR	−35.1 (−54.6–53.3)	0.1 (−16.6–39.0)	4.3 (−21.9–39.9)	5.9 (−32.6–39.7)	0.18	0.03	0.76	0.70
Wrist ROM pro/supination	37.0 (17.0–89.9)	74.5 (35.0–114.0)	69.0 (1.0–144.0)	144.5 (75.0–165.0)	0.0024	0.0391	0.002	0.0098
Thoracic abduction at PAR	1.3 (−8.0–18.7)	−1.6 (−6.9–2.1)	−1.6 (−13.4–5.4)	−3.1 (−37.4–3.0)	0.3652	0.0840	0.24	0.0840
Thoracic abduction at max. elbow flexion	3.2 (−18.7–17.0)	0.1 (−6.4–8.8)	−1.0 (−17.0–12.2)	−2.7 (−33.3–2.6)	0.8984	0.8457	0.24	0.16
Thoracic flexion at PAR	5.3 (−74.9–39.4)	6.4 (−0.1–34.1)	−2.2 (−17.0–9.6)	−0.9 (−15.3–14.6)	0.2676	0.4143	0.14	0.0681
Thoracic flexion at max. elbow flexion	−9.2 (−54.0–10.6)	3.4 (−16.8–19.6)	−4.3 (−17.0–12.7)	−2.7 (−15.3–12.7)	0.0006	0.1909	0.50	0.38
Thoracic rotation at PAR	−1.7 (−18.5–6.2)	−2.5 (−31.0–10.4)	−9.4 (−88.9–86.3)	−3.4 (−89.9–88.2)	0.9032	0.2163	0.90	0.64
Thoracic rotation at max. elbow flexion	1.6 (−35.9–27.7)	0.4 (−16.1–5.1)	−9.7 (−85.6–88.4)	−2.8 (−89.7–88.2)	0.5016	0.3054	0.76	0.50

All included data presented as median and range in angular degrees, or degrees/ms (*). OP: operatively; PAR: position at rest; ROM: range of motion.

## Data Availability

Detailed data supporting the results are available from the authors.

## Supplementary Materials

The following supporting information can be downloaded at: https://www.mdpi.com/article/10.3390/jcm13102759/s1, Figure S1: Pre- and post-operative elbow mobility; Figure S2: Pre- and post-operative thoracic mobility.
