# Supplementary material for: Kinematic Effects of Derotational Osteotomy of the Humerus in Patients with Internal Shoulder Contracture Secondary to Erb’s Palsy—A Retrospective Cohort Study
Source: J Clin Med. 2024 May 8;13(10):2759. doi: 10.3390/jcm13102759 (PMC11121948; doi:10.3390/jcm13102759)
Supplement: Supplementary file 1 [file jcm-13-02759-s001.zip › jcm-2943498-supplementary.pdf]

Supplementary Materials

Kinematic Effects of Derotational Osteotomy of the Humerus in Patients with Internal Shoulder Contractures Secondary to Erb's Palsy

A Prospective Cohort Study

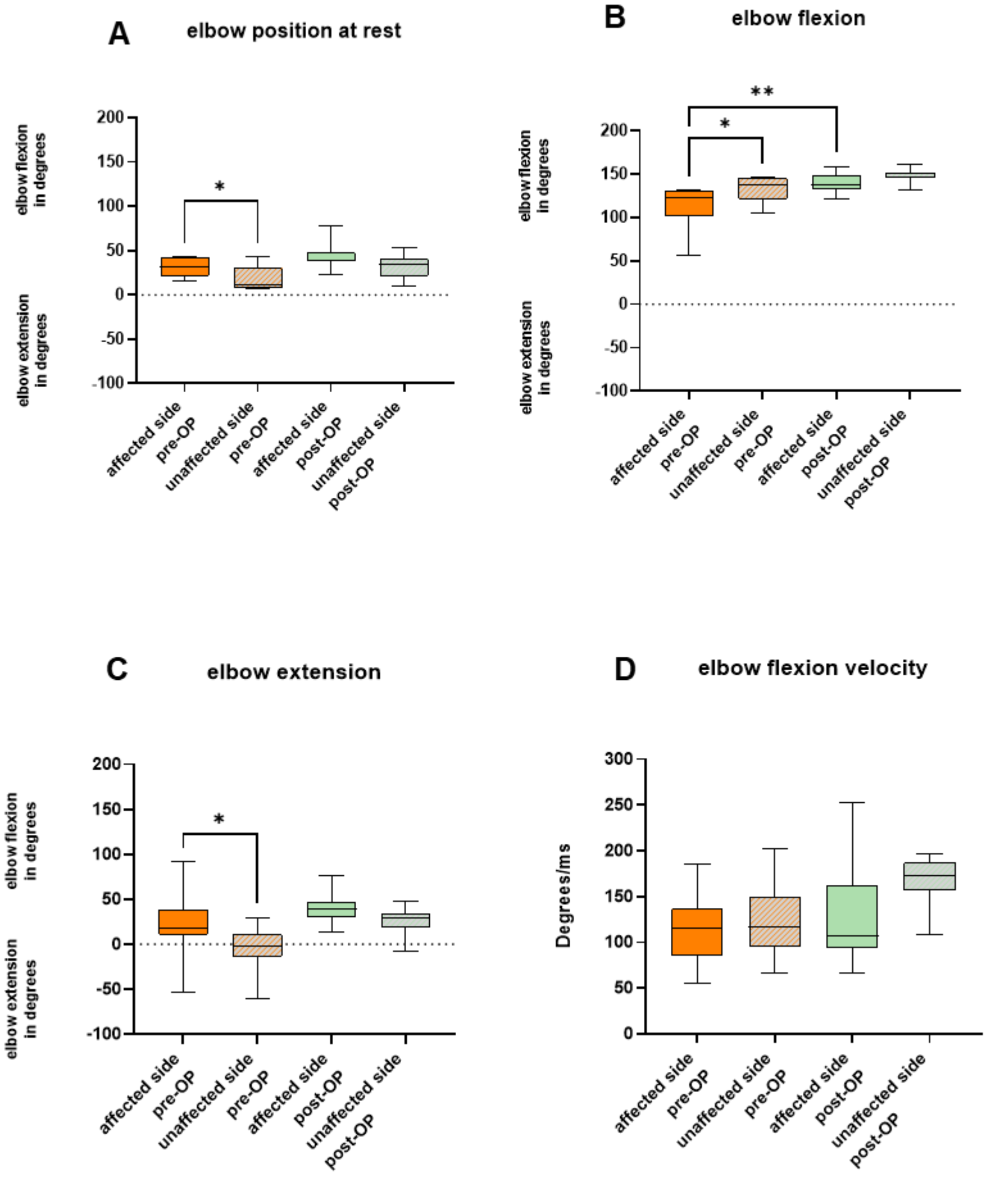

Figure S1. Pre- and post-operative elbow mobility: (A) The box plots illustrate angular degrees of the elbow position at rest of the affected and unaffected limb both pre- and post-operatively. (A) and (B) show the angular degrees achieved with the affected and unaffected limb both pre- and postoperatively when patients were asked to perform (B) maximum elbow flexion and (C) maximum elbow extension. (D) Highest possible velocity of elbow flexion in degrees/ms achieved with the affected and unaffected limb both pre and postoperatively. The whiskers extent to the minimum and maximum values. * = $p < 0.05$; ** = $p < 0.01$.

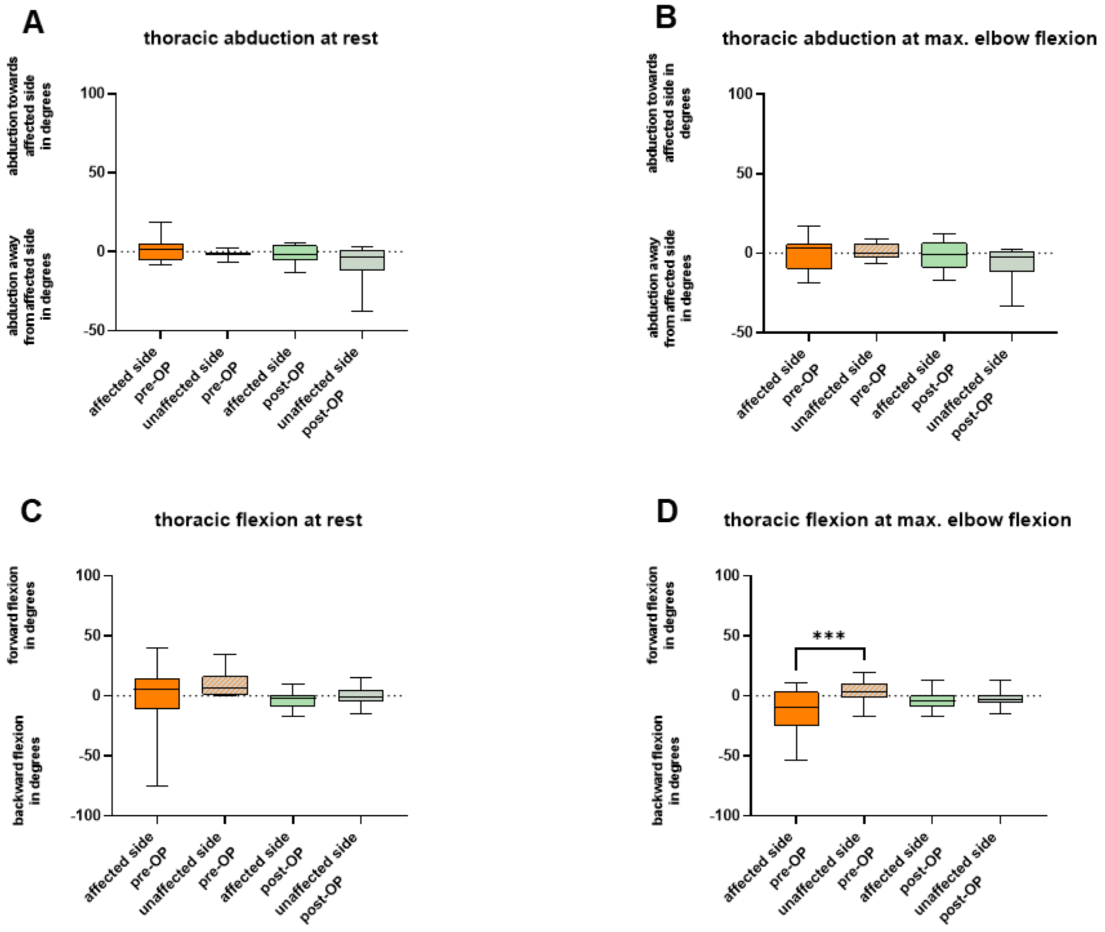

Figure S2. Pre- and post-operative thoracic mobility: (A) The box plots show the amount of abduction of the trunk towards and away from the affected side of the body in the position at rest. Positive values indicate thoracic abduction towards the affected side, while negative values indicate thoracic abduction away from the affected side. (B) shows the thoracic abduction when patients performed maximum elbow flexion. (C) shows the angular degrees of thoracic flexion at the position at rest. Positive values indicate thoracic flexion forward, while negative values indicate thoracic flexion backward. (D) shows the thoracic flexion when patients performed maximum elbow flexion. The whiskers extent to the minimum and maximum values. *** = $p < 0.001$.
